# Native Aortic Valve Endocarditis Complicated by Splenic Infarction and Giant Mitral-Aortic Intervalvular Fibrosa Pseudoaneurysm—A Case Report and Brief Review of the Literature

**DOI:** 10.3390/diagnostics11020251

**Published:** 2021-02-06

**Authors:** Andreea Varga, Ioan Tilea, Cristina Maria Tatar, Dragos Gabriel Iancu, Maria Andrada Jiga, Robert Adrian Dumbrava, Marian Pop, Horatiu Suciu

**Affiliations:** 1 Department ME2, Faculty of Medicine in English, “G. E. Palade” University of Medicine, Pharmacy, Science and Technology of Targu Mures, 540142 Targu Mures, Romania; andreea.varga@umfst.ro; 2 Department of Cardiology II, Emergency Clinical County Hospital, 540042 Targu Mures, Romania; ioan.tilea@umfst.ro; 3 Department M4, Clinical Sciences, Faculty of Medicine, “G. E. Palade” University of Medicine, Pharmacy, Science and Technology of Targu Mures, 540142 Targu Mures, Romania; 4 Department of Internal Medicine II, Emergency Clinical County Hospital, 540042 Targu Mures, Romania; dragos-gabriel.iancu@umfst.ro (D.G.I.); maria-andrada.jiga@umfst.ro (M.A.J.); robert-adrian.dumbrava@umfst.ro (R.A.D.); 5 Doctoral School, “G. E. Palade” University of Medicine, Pharmacy, Science and Technology of Targu Mures, 540142 Targu Mures, Romania; 6 Department ME1, Faculty of Medicine in English, “G. E. Palade” University of Medicine, Pharmacy, Science and Technology of Targu Mures, 540142 Targu Mures, Romania; marian.pop@umfst.ro; 7 Department of Radiology and Medical Imaging, The Emergency Institute for Cardiovascular Diseases and Transplantation, 540136 Targu Mures, Romania; 8 Department M3, Clinical and Surgery Sciences, Faculty of Medicine, “G. E. Palade” University of Medicine, Pharmacy, Science and Technology of Targu Mures, 540142 Targu Mures, Romania; horatiu.suciu@umfst.ro; 9 Cardiac Surgery Clinic, The Emergency Institute for Cardiovascular Diseases and Transplantation, 540136 Targu Mures, Romania

**Keywords:** bicuspid aortic valve, infective endocarditis, mitral-aortic intervalvular fibrosa pseudoaneurysm, splenic infarction, management

## Abstract

Background: Pseudoaneurysm of the mitral-aortic intervalvular fibrosa (P-MAIVF) is an unusual complication related to various injuries or conditions which involve the mitro-aortic region; it communicates with the left ventricular outflow tract and is associated with a high-risk of redoubtable complications or sudden death. The cerebral and splenic localizations are frequently seen as manifestations of systemic embolism in infective endocarditis. Currently, there are no specific recommendations related to the diagnosis, management, treatment, or further evolution of patients with P-MAIVF and concomitant splenic infarction. This paper presents the case of a 43-year-old Caucasian woman with a late diagnosis of mixed bicuspid aortic valve disease, affected by an under-detected and undertreated episode of infective endocarditis leading to asymptomatic P-MAIVF. Prime clinical and imagistic diagnosis of splenic infarction indicated further extended investigations were required to clarify the source of embolism. Methods: Integrated multimodality imaging techniques confirmed the unexpected diagnosis of P-MAIVF. Results: The case had a fatal outcome following an uncomplicated yet laborious cardiac surgery. Patient death was attributed to a malignant ventricular arrhythmia. Conclusion: The present case raises awareness by highlighting an unexplained and unexpected splenic infarction association with P-MAIVF as a result of infective endocarditis related to mixed bicuspid aortic valve disease.

## 1. Introduction

The mitral-aortic intervalvular fibrosa (MAIVF) is an avascular frail fibrous structure that delineates the anterior mitral leaflet from the posterior portion of the aortic root. It is bounded by the pericardium in the upper part, and the left atrium in the posterolateral part; the left ventricular outflow tract (LVOT) represents the lower limit of the MAIVF [1,2]. Pseudoaneurysm of the mitral-aortic intervalvular fibrosa (P-MAIVF), first described by Waldhausen et al., usually ensues as a consequence of infective endocarditis (IE) or valve surgery. Congenital heart diseases, direct thoracic injuries, Takayasu arteritis, and other cardiac interventional maneuvers can result in several severe complications [3,4,5,6]. Perforation secondary to infection of the mitral-aortic intervalvular fibrosa can lead to the development of P-MAIVF, particularly in patients with a bicuspid aortic valve, due to congenital weakness of this area, as described by Qizilbash et al. [7].

Complications of a P-MAIVF can lead to its rupture in the left atrium with the onset of acute heart failure or to the pericardium with the appearance of cardiac tamponade and sudden death [8]. Angina or myocardial infarction due to the compression of coronary arteries (frequently left anterior descending artery) and compression of pulmonary arteries have also been described [8,9,10,11]. Approximately 12% of cases experience cerebrovascular or embolic events, perhaps after clot formation in the pseudoaneurysm [12,13,14].

Hitherto, comprehensive data related to clinical presentation, laboratory tests, imagistic studies, management, and outcomes are limited as a result of the low-frequency of P-MAIVF reported cases [10,15,16].

Splenic infarcts (SI) occur as a result of embolization in 5.4% of patients with left-sided infective endocarditis; they can evolve to abscess formation in approximately 5% of patients [17,18,19]. In the present paper, we report a large P-MAIVF complicated with SI and abscess in a middle-aged Caucasian woman with negative blood cultures infective endocarditis affecting the bicuspid aortic valve.

## 2. Case Presentation

A 43-year-old Caucasian woman was diagnosed with bicuspid aortic valve and severe valvular aortic stenosis during full-term pregnancy (aged 27-years in 2004). Aortic valve replacement was shunned for fear of intervention; without cardiological follow-up, she was admitted in February 2020 complaining of intense and prolonged pain in the left hypochondrium, fatigue, and dyspnea, with acute onset three days before presentation.

On admission, her vitals indicated no fever, a heart rate of 87 bpm, blood pressure of 100/70 mmHg, and values for peripheral oxygen saturation were not outside the normal range. Physical examination revealed facial hyperemia, bilateral limb edema, systolic aortic murmur, and mild tenderness in the left hypochondriac region.

Laboratory workup indicated the expected values were within normal limits, with the exception of mild thrombocytopenia (132.7 × 10^3^/µL, normal range 150–400 × 10^3^/µL), low serum iron (8.2 µmol/L, normal levels: 11.6–31.3 µmol/L), serum ferritin of 68 ng/mL, moderately elevated triglycerides (2.22 mmol/L, normal range: 0.55–1.9 mmol/L), and positive C-reactive protein (CRP). The baseline NT-proBNP value was 1442 pg/mL. Subsequently, serologic patterns suggestive for hepatitis B surface antigen or antibodies for C hepatitis, nasal, pharyngeal, and serial blood culture tests were negative for bacteria and fungi.

Rest electrocardiograms and 24 h Holter ECG recordings displayed left axial deviation, signs of left ventricular hypertrophy (LVH), and rare, isolated premature ventricular contractions.

**Figure 1:**
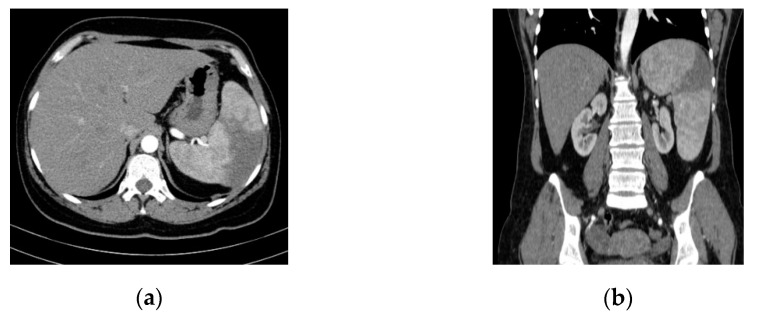
Abdominal/pelvic contrast-enhanced CT scan (Optiray™ 350). Splenomegaly and splenic infarction are depicted in (**a**) axial and (**b**) coronal sections.

An abdominal ultrasound examination (HS60 ultrasound machine, Samsung Medison Co., Ltd., Seoul, Korea) and CT study (Somatom Emotion™ 16-slice, Siemens Healthcare GmbH, Erlangen, Germany) highlighted splenomegaly and splenic infarction (Figure 1).

**Figure 2:**
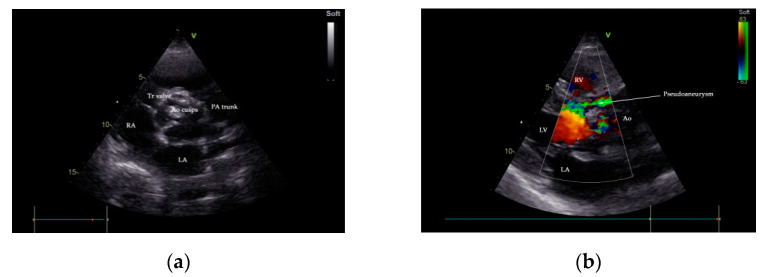
Transthoracic echocardiography (TTE), parasternal short-axis-base view, showing a heavily calcified aortic valve with possible vegetation (**a**); communication between LVOT and P-MAIVF is shown by color flow Doppler, parasternal-long axis view (**b**). Ao—aorta, LA—left atrium, LVOT—left ventricular outflow tract, PA—pulmonary artery, P-MAIVF—pseudoaneurysm of the mitral-aortic intervalvular fibrosa, RA—right atrium, RV—right ventricle, Tr—tricuspid valve.

**Figure 3:**
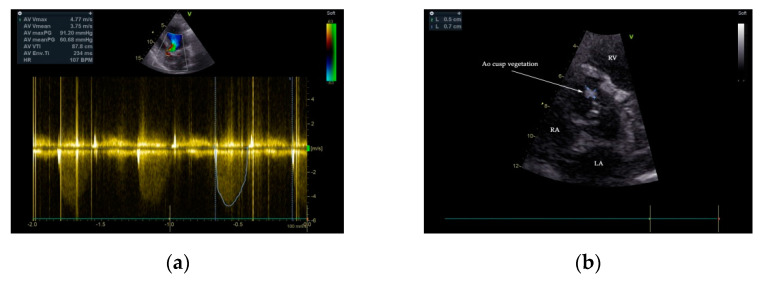
TTE 2D color flow and continuous Doppler 2D examination demonstrated severe aortic stenosis—aortic valve gradient 91/60 mmHg (**a**). The presence of a vegetation of 5 mm × 7 mm attached to the anterior cusp of the aortic valve is depicted (**b**).

Two-dimensional transthoracic and transesophageal echocardiography (TTE and TOE, respectively) were performed (GE Vivid^TM^ E9 ultrasound system, GE Healthcare, Boston, MA, USA). Analysis of TTE images denoted a large P-MAIVF (60 mm × 6 mm), LVH, normal dimensions of the left ventricle with an LV ejection fraction of 65% (modified Simpson’s biplane method), bicuspid aortic valve, and severe aortic stenosis (peak velocity: 4.8 m/s, gradient of 91/60 mm Hg, AVA: 0.51 cm^2^/m^2^). Mild aortic and mitral regurgitation were noted. A 5 mm × 7 mm vegetation was identified at the level on the anterior aortic cusp (Figure 2 and Figure 3). Echocardiographic assessment did not confirm pericardial effusion or the presence of pulmonary hypertension (Supplementary Movie S1).

**Figure 4:**
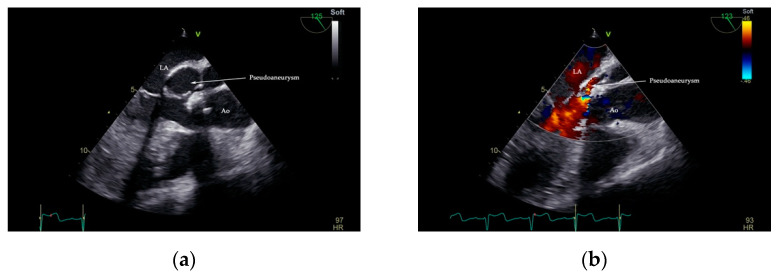
Two-dimensional TOE showing the presence of pseudoaneurysm of the mitral-aortic intervalvular fibrosa (P-MAIVF). Arrow—pseudoaneurysm lumen in systole (**a**). A turbulent flow seen in the pseudoaneurysm lumen during diastole (**b**). Ao—aorta, LA—left atrium.

Interpretation of TOE images digitally archived in DICOM format confirmed the presence of an echo-lucent space that displayed a characteristic systolic expansion and a diastolic collapse, and a posterior aortic-oriented communication with LVOT between the left atrium, mitral valve, and aortic valve. Color flow Doppler visualized a highly turbulent flow in the pseudoaneurysm (Figure 4, Supplementary Movie S2, and S2.1).

**Figure 5:**
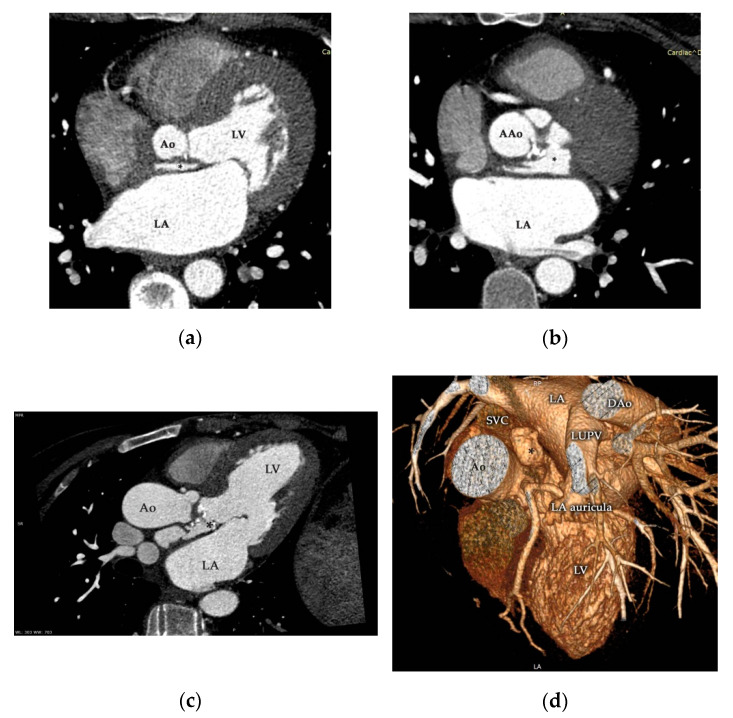
Contrast-enhanced ECG-gated multidetector-row cardiac computed tomography (MDCT) study of the aortic valve and ascending aorta during the cardiac cycle corroborated the presence of P-MAIVF (*) with the appearance of contrast media (**a**–**c**). Three-dimensional reconstruction (**d**) supported the aforementioned findings, and precise localization is clearly demonstrated. Ao—aorta, AAo—ascending aorta, DAo—descending aorta, LA—left atrium, LUPV—left upper pulmonary vein, LV—left ventricle, SVC—superior vena cava.

Contrast-enhanced ECG-gated multidetector-row cardiac computed tomography (MDCT; Somatom Definition Flash™, Siemens Healthcare GmbH, Erlangen, Germany) corroborated the aforementioned presence of a giant pseudoaneurysm originating in the LVOT at the level of the mitral and aortic intervalvular fibrous fascia extending cranially between the right pulmonary artery and the anterior caudal of the left atrium (Figure 5, Supplementary Movie S3). Coronary arteries were normal, with no signs of compression and the absolute coronary artery calcium score (Agatston method) was zero.

Pharmacological medication (spironolactone, furosemide, metoprolol, and antibiotics) was initiated. The antibiotic regimen was prescribed according to the current guidelines [18]. Intravenous iron (ferric carboxymaltose) was offered to correct iron deficiency anemia. The patient was referred to cardiac surgery. In total cardiopulmonary bypass and moderate hypothermia, Morrow classical septal myectomy was performed; because the aneurysm itself was not technically amenable to resection, and the aortic wall had sufficient structural integrity, the surgical team decided to suture the ostium of the P-MAIVF. Surgery was completed by replacement of the affected valve with a standard St. Jude Medical 19 (St Jude Medical Inc., St Paul, MN, USA) mechanical valve prosthesis.

The postoperative course was uneventful for the first week; the patient suddenly succumbed before discharge and died on day ten. Her death was attributed to a malignant ventricular arrhythmia.

## 3. Discussion

Complications of infective endocarditis are numerous, unpredictable, and severe. In our case, we diagnosed a pseudoaneurysm of the mitral-aortic intervalvular fibrosa, culture-negative endocarditis, severe aortic stenosis, moderate aortic and mitral regurgitation, heart failure with preserved ejection fraction NYHA functional class III, splenic infarction, iron deficiency anemia, and thrombocytopenia. The literature includes few reports on the coexistence of more than one mechanical complication of IE. Multimodality imaging techniques are used to identify and describe these complications.

As previously mentioned, P-MAIVF commonly occurs after aortic and mitral valve replacement surgery, direct thoracic injuries in intravenous drug-dependence subjects, or, notably, in patients with prosthetic aortic valve IE [5,8,12,15,16,20,21,22,23,24]. However, we present a case of P-MAIVF related to mixed bicuspid aortic valve (MBAV) disease infective endocarditis.

A broad-spectrum of clinical manifestations, from asymptomatic to critical cardiac tamponade resulting from pseudoaneurysm rupture, can be observed [7,8,18]. In our patient, marked fatigue, progressive dyspnea, and violent abdominal pain were recognized.

Transesophageal echocardiography has better sensitivity than TTE in the diagnosis of vegetations in native and prosthetic valves, abscesses, pseudoaneurysms, and other mechanical complications of heterologous cardiac valves in IE patients [25]. TOE is recognized to be the modality of choice for P-MAIVF diagnosis in selected cases. Multiplane examination discerns the typical anatomical site and complexity of P-MAIVF anatomy, systolic expansion with flow evidence from LVOT into the pouch, and diastolic flow from the aneurysm to LVOT, causing a collapse of the cavity [12,26]. Aortocavitary fistulous tracts are detected in up to 97% of cases using TOE [26]. In our case, the suspicion of P-MAIVF was raised by TTE and confirmed by TOE and the angiographic CT chest scan.

Xie et al. reviewed 149 cases of P-MAIVF from studies published from 1966 to 2012. Causative organisms included *Staphylococcus aureus*, various *Streptococcus* spp., *Mycobacterium tuberculosis*, *Enterococcus* spp., *Brucella suis*, *Pecilomyces lilacinus*, *Monilia albicans*, and *Bacillus* spp. [13]. According to this review, there were no organism-specific characteristics in the occurrence of P-MAIVF. Serial sets of blood cultures in our case remained negative during hospitalization.

Intra-abdominal embolic complications are relatively common in cases of left-sided infective endocarditis. The spleen and the brain are the most frequent sites of systemic embolism [27]. Jasarevic et al. reviewing abdominal angiographic CT studies, suggested that splenic infarction may be suspected in the presence of a consistently triangular-shaped area with hypodensity on portal venous phase images, with no late enhancement [28]. In our case, SI was suspected due to the presence of intense and prolonged pain in the splenic lodge as well as abdominal ultrasonography and was confirmed by abdominal CT scan. No extra-splenic embolic lesions (kidney, liver) related to IE were detected.

Management of uncomplicated pseudoaneurysms of the mitral-aortic intervalvular fibrosa is not well-defined; in asymptomatic cases or in patients who do not undergo surgery, a careful clinical and echocardiographic follow-up with watchful judgment is particularly valid [29,30,31]. The possibility of the development of life-threatening conditions is significant; thus, after careful case-by-case assessment, early surgical intervention is the cornerstone treatment for reducing mortality [14]. Published papers have described aortic valve replacement alone or combined with individualized reconstruction procedures (i.e., simple aneurysmal suture or reinforced with various materials, homograft aortic root replacement, and coronary artery bypass grafting) as of current open-surgery options [13,14,15]. Few authors have described successful percutaneous device-therapy of P-MAIVF using various devices (coil embolization, septal occluder devices, vascular plugs, transapical implantation of an Edwards Sapien XT valve-in-valve fashion) as an alternative when surgery is not appropriate [32,33,34]. Recently, Boi et al. reported the first successfully exclusion of a P-MAIVF by transapical transcatheter implantation of a balloon-expandable Edwards Sapien 3 Ultra valve in a 78-year-old man with concomitant severe aortic stenosis [35].

Complete resolution of a medically treated P-MAIVF is extremely rare; in 2005, Ghersin et al. presented a case of a 61-year-old woman with severe bicuspid aortic stenosis diagnosed with a P-MAIVF and IE developed 9 months after aortic valve replacement with a bileaflet mechanical prosthesis. The case was treated with systemic antibiotics, and dynamic serial imaging studies (MDCT, TOE) performed 10 months after the initial diagnosis was conclusive in the dimensions of the P-MAIVF remaining unchanged with a reduction in vegetation [36].

Further studies identifying possible inflammatory factors, such as cytokines, as predictable markers of cardiovascular pathology could increase early diagnosis of individual cases [37]. It is already demonstrated that lower levels of chemerin in patients with severe aortic stenosis are associated with the cessation of inflammatory processes [38].

In our case, we scheduled cardiac surgery due to the presence of symptomatic aortic valve disease leading to heart failure and the large size of the pseudoaneurysm with the presence of a fistula, which is known to be a high-risk feature for progression of complications.

## 4. Conclusions

The existence of late complications of insufficiently treated infective endocarditis in a middle-aged woman who experienced a delayed diagnosis of mixed bicuspid aortic valve disease highlights the importance of awareness of P-MAIVF and splenic infarction. This high-risk association should be considered.

In summary, our case underscores the value of integrated multimodality imaging techniques as a component of timely diagnostic frameworks that are essential to the formulation of appropriate strategic decisions in assessing a pseudoaneurysm of the mitral aortic intervalvular fibrosa secondary to infective endocarditis of a native aortic valve.

## Data Availability

The data presented in this paper are available on request from the corresponding author. The data are not publicly available due to national and local general data protection regulations.

## Supplementary Materials

The following are available online at https://www.mdpi.com/2075-4418/11/2/251/s1.
